# Molecular Mechanisms of Cardiomyocyte Death in Drug-Induced Cardiotoxicity

**DOI:** 10.3389/fcell.2020.00434

**Published:** 2020-06-03

**Authors:** Wanjun Ma, Shanshan Wei, Bikui Zhang, Wenqun Li

**Affiliations:** ^1^Department of Pharmacy, The Second Xiangya Hospital, Central South University, Changsha, China; ^2^Institute of Clinical Pharmacy, Central South University, Changsha, China

**Keywords:** cardiotoxicity, cardiomyocytes, cell death, apoptosis, autophagy, necrosis

## Abstract

Homeostatic regulation of cardiomyocytes plays a crucial role in maintaining the normal physiological activity of cardiac tissue. Severe cardiotoxicity results in cardiac diseases including but not limited to arrhythmia, myocardial infarction and myocardial hypertrophy. Drug-induced cardiotoxicity limits or forbids further use of the implicated drugs. Such drugs that are currently available in the clinic include anti-tumor drugs (doxorubicin, cisplatin, trastuzumab, etc.), antidiabetic drugs (rosiglitazone and pioglitazone), and an antiviral drug (zidovudine). This review focused on cardiomyocyte death forms and related mechanisms underlying clinical drug-induced cardiotoxicity, including apoptosis, autophagy, necrosis, necroptosis, pryoptosis, and ferroptosis. The key proteins involved in cardiomyocyte death signaling were discussed and evaluated, aiming to provide a theoretical basis and target for the prevention and treatment of drug-induced cardiotoxicity in the clinical practice.

## Introduction

Cardiotoxicity commonly refers to toxicity that has a detrimental impact on the heart, which might finally lead to myocardiopathy such as arrhythmia, myocardial infarction and myocardial hypertrophy. These inevitable side effects, especially of anticancer drugs, are usually the main causes of treatment termination and drug development failure. In addition, modern cancer treatments recommend a combination of multiple agents, almost always leading to synergistic side-effects (Ewer and Ewer, 2015). In the past few decades, more than 10% of clinical drugs were forced out of the market due to cardiovascular side effects, which still hindered the drug development and seriously affected the improvement of patient health. Many studies have revealed that drug-induced myocardial damage may be a stepwise process accompanied by the increase of cardiac biomarkers and structural myocardial deformation, finally resulting in left ventricular ejection fraction (LVEF) decrease (Pistillucci et al., 2015; Patel and Cornell, 2019). Currently, the widely accepted definition of cardiotoxicity is the decline in LVEF of at least 10% to less than 55% (Nicol et al., 2019). Clinical data show that cardiomyocyte death or damage concomitantly takes place with the progression of cardiotoxicity, indicating that drug-induced cardiomyocyte death may be the main cause of cardiotoxicity.

## Overview of Cell Death Forms

It is widely accepted that cell death, proliferation and differentiation are essential throughout the pathological and physiological processes. Although more than ten types of cell death have been discovered to date, the most common forms of cell death in drug-induced cardiotoxicity are apoptosis, autophagy and necrosis (Galluzzi et al., 2018). In addition, recently discovered cell death forms, such as necroptosis, pyroptosis and ferroptosis, are also involved in drug-induced cardiotoxicity.

### Apoptosis

Morphologically, apoptosis is the most widely studied cell death form, exhibiting signs of cell shrinkage, increased cytoplasmic density, decreased mitochondrial membrane potential (MMP) disappearance and changes in permeability. Eventually, intact apoptotic bodies are formed to be efficiently absorbed and degraded by adjacent cells. Based on the underlying mechanisms, apoptosis is divided into intrinsic and extrinsic apoptosis. Intrinsic apoptosis is caused by microenvironment disorders such as DNA damage, excessive oxidative stress, mitotic disaster, loss of growth factor signaling and endoplasmic reticulum (ER) stress (Brumatti et al., 2010; Wu and Bratton, 2013; Czabotar et al., 2014; Roos et al., 2016). B cell lymphoma-2 (Bcl-2) family pro-apoptotic members, such as Bax, Bak, and BH3-only protein, primarily regulate the intrinsic cell apoptosis via their influence on mitochondria. Bcl-2 stimulates mitochondria translocation of Bax/Bak and causes mitochondrial membrane permeabilization, which eventually leads to the release of cytochrome C in the cytoplasm, where the apoptosome forms and caspases cascade reactions arise (Hutt, 2015). Unlike intrinsic apoptosis, the extrinsic apoptosis is mainly initiated by two kinds of plasma membrane receptors including Fas cell surface death receptor (Fas) and tumor necrosis factor receptor (TNF) super family member (TNFR1, TNFRSF10A, and TNFRSF10B) along with their respective homologous ligands. Death-inducing signaling complex (DISC), composed of death ligands and corresponding receptors on the cell membrane, is a receptor proximal protein complex that helps connect the death receptor signaling with caspases cascade reactions. The pro-apoptotic proteins caspase-8 and caspase-10 are recruited and cleaved by upstream caspase enzyme to initiate apoptosis (Barnhart et al., 2003; Yang, 2015).

### Autophagy

As a pro-survival mechanism, autophagy occurs in destroying and recovering unwanted or damaged cellular components, playing an essential role in maintaining intracellular metabolic homeostasis (Yang et al., 2017). A series of evidences suggest that the autophagy-activating kinase 1 (ULK-1) initiates autophagy by phosphorylating Beclin1 and activating the vacuolar protein sorting 34 (VPS34) complex (Klionsky et al., 2016). As a critical signaling protein of autophagy, the mammalian target of rapamycin (mTOR) can be activated by nutritional deficiency, growth factor and receptor tyrosine kinase, which subsequently forms mammalian target of rapamycin complex 1 (mTORC1) with several other proteins. The mTORC1 further binds to the ULK-1 complex and blocks ULK-1-mediated Beclin 1 phosphorylation, thus inhibiting autophagy initiation (Jung et al., 2010). In contrast, the adenosine 5-monophosphate activated protein kinase (AMPK) mediates autophagy by decreasing mTOR-related autophagy suppression and phosphorylating the ULK-1 complex at Ser317 and Ser777 (Kim et al., 2011).

### Necrosis

In the past few decades, necrosis is typically described as a form of passive and irreversible cell death that is always associated with pathology, usually accompanied by morphological characteristics such as increased membrane permeability, disintegration of organelles and cell swelling. As a traditional cell death form, necrosis occurs usually after the exposure to extreme physical or chemical insults, and therefore is regarded as an accident and unregulated cell death form. With in-depth study, more regulated cell death forms are proposed, including necroptosis, pryoptosis and ferroptosis, which share similar morphological characteristics with necrosis.

### Other Regulated Cell Death Forms: Necroptosis, Pryoptosis, and Ferroptosis

In contrast to necrosis, necroptosis is regulated by specific transduction mechanism. Death receptor TNFR1 plays a key role in the development of necroptosis (Kaiser et al., 2013). Activation of TNFR1 can stimulate RIPK1 to further recruit RIPK3 that leads to necrosomes formation (Grootjans et al., 2017). In addition, sequential activation of RIPK3/MLKL is also crucial in necroptosis signaling (Song and Wang, 2013). Phosphorylated MLKL can destroy the plasma membrane and organelles to release inflammatory factors, and elicit an immune response, indicating the occurrence of necroptosis (Galluzzi et al., 2018). It is worth mentioning that caspase-8 plays a suppressing role in necroptosis since it inactivates RIPK1 and RIPK3 (Belmonte et al., 2015; Tummers and Green, 2017).

Pyroptosis, firstly proposed by Cooksonis, is widely recognized as inflammatory and regulated cell death form that usually occurs in defense of exogenous pathogens such as virus, bacteria and fungi (Cookson and Brennan, 2001; Jorgensen and Miao, 2015). Activation of caspases including caspase-1, caspase-3, caspase-4 and caspase-11 is necessary for initiating the pyroptosis, which further specifically cleaves GSDMD or GSDME to generate holes in the membrane and release interleukin-1beta (IL-1β) and IL-18, inducing pyroptosis (Shi et al., 2015; Man et al., 2017). Recent studies have identified a potential relationship between pyroptosis and myocardial injury (Chen et al., 2018).

Unlike pyroptosis, ferroptosis is firstly discovered in carcinoma cells and characterized by the accumulation of iron and lipid reactive oxygen species (ROS), which could deplete anti-oxidases and cause mitochondrial damages, leading to cell death (Sumneang et al., 2020). Moreover, Chen et al. (2019) found that the inhibition of Toll like receptor 4 (TLR4) and triphosphopyridine nucleotide oxidase 4 (NOX4) significantly alleviated ferroptosis. Glutathione peroxidase 4 (GPx4) can prevent erastin and RSL3-induced ferroptosis via suppressing lipid peroxidation (Imai et al., 2017).

## Cardiomyocyte Death in Drug-Induced Cardiotoxicity

**FIGURE 1:**
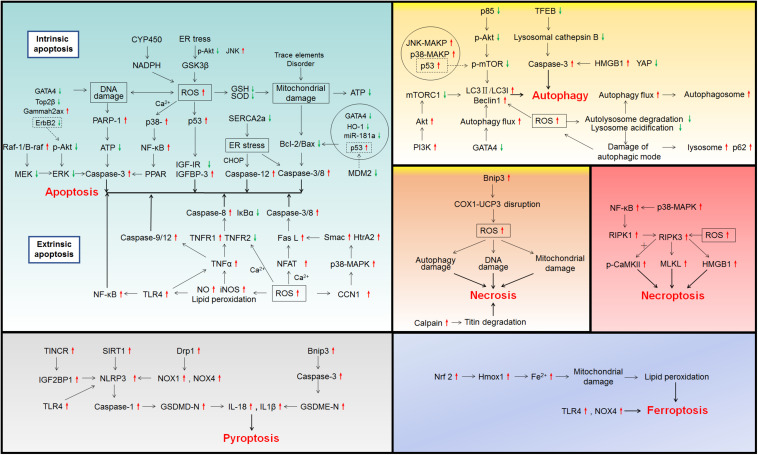
Signaling pathways involved in drug-induced cardiotoxicity.

Multiple evidences have suggested that there is a strong correlation between drug-induced cardiomyocyte death and cardiotoxicity. Here, we will summarize and discuss cardiomyocyte death induced by the drugs listed in Table 1 and their underlying cell death mechanisms shown in Figure 1.

**TABLE 1:** Molecular mechanisms of cardiomyocyte death in drug-induced cardiotoxicity.

**Drug type**	**Drugs**	**Cell death type**	**Cell type/animal**	**Mechanism**	**References**
Anticancer drug	Doxorubicin	Apoptosis	C57BL/6J mice	Cytochrome P450-NADPH-ROS↑-DNA damage	Deng et al., 2007
			Adult cardiac myocytes, HL-1 cardiac muscle cells	GATA4↓-DNA damage	Kim et al., 2003
			Cardiomyocytes; C57BL/6J mice	ROS↑, RNS↑-PARP-1-ATP↓	Mukhopadhyay et al., 2009
			Sprague Dawley (SD) rats	Fe↑-ROS↑-Bcl-2/Bax↓-caspase-3↑, Cytochrome C↑	Childs et al., 2002
			Cardiomyocytes; Mice	Top2β-DNA damage-ROS↑	Zhang et al., 2012
			Pluripotent stem cells-derived cardiomyocytes	TNFR1↑, Fas↑, DR4↑, DR5↑, TRAIL↑	Zhao and Zhang, 2017
			Adult rat cardiomyocytes, Rat H9c2 cardiac cells	ROS↑-Ca^2+^↑-NFAT↑- Fas L↑- caspase-3/8↑	Kalivendi et al., 2005
			Neonatal rat cardiomyocytes, cardiac fibroblasts; C57/B6 mice	CFs-Fas L exosome release- cardiomyocyte	Zhang et al., 2019
			Primary cardiomyocytes	Fas L↑-caspase-8↑	Yamaoka et al., 2000
			Rat cardiomyoblast H9c2 cells; C57BL/6 mice	ROS↑-CCN1↑-p38-MAPK↑-Smac↑, HtrA2↑-Fas L↑	Hsu and Mo, 2016
			H9c2 cells	TNFα↑-TNFR1↑, TNFR2↓-caspase-8↑, IκBα↓	Chiosi et al., 2007
			Male Wistar rats	ROS↑-NO↑, iNOS↑, Lipid peroxidation-TNFα↑, IL-1β↑, IL-6↑	Akolkar et al., 2017
			SD rats	Ca^2+^↑-TNFα↑-caspase-9/12↑	Agustini et al., 2016
			Adult Wistar rats	ROS↑-mitochondrial damage	Giulivi et al., 1995
			Neonatal cardiac myocytes	Mn-SOD↓-MMP↓-Bcl-2↓, Bax↑, Cytochrome C↑	Chae et al., 2005
			Kunming mice	Mn-SOD↓-MMP↓, MPT-caspase↑	Li W. J. et al., 2018
			NRCMs; C57BL/6 mice	miR-146a↑-ErbB4↓	Horie et al., 2010
			Primary cardiomyocytes, H9c2 cells	miR-181a↓-Bcl-2↓	Zhao et al., 2018
			H9c2 cells	HO-1↓-Bax↑, Cytochrome C↑	Bernuzzi et al., 2009
			Male wild-type Balb/c mice	p53↑-Bax↑, Cytochrome C↑	Zhang et al., 2011
			H9c2 cells	ROS↑, p53↑-IGF-IR↓, IGFBP-3↑	Fabbi et al., 2015
		Autophagy	H9c2 cells; C57BL/6J mice	ROS↑-LC3II/LC3I↑, Beclin1↑	Zhang et al., 2015
			Adult rat cardiomyocytes	p53↑, p38-MAKP↑, JNK-MAKP↑	Ludke et al., 2017
			Kunming mice	p85↓-phosphorylated AKT↓-phosphorylated mTOR↓	Yu et al., 2017
			MHC-CB7 mice	p53↑-phosphorylated mTOR↓	Zhu et al., 2009
			Adult rat ventricular myocytes (ARVMs); Male Wistar rats	ROS↑-lysosome acidification↓-autophagy flux↑, autophagosomes↑	Dimitrakis et al., 2012
			ARVMs, NRCMs, H9 human embryonic stem Cells induced cardiomyocytes; C57BL/6 mice	lysosome acidification↓, autolysosome degradation↓-damage of autophagic mode	Li et al., 2016
			NRCMs, adult rat cardiomyocytes, adult mouse cardiomyocytes, H9c2 rat embryonic cardiomyoblasts; C57BL/6J mice, SD rats	TFEB↓-lysosomal cathepsin B↓-caspase-3↑	Bartlett et al., 2016
			NRCMs; Wistar rats	HMGB1↑, YAP↓-caspase-3↑	Luo et al., 2018
			NRCMs	GATA4↓-autophagy flux↑-Bcl-2↓, Beclin 1↑	Kobayashi et al., 2010
		Necrosis	Cardiomyocytes; C57BL/6J mice	ROS↑, RNS↑	Mukhopadhyay et al., 2009
			H9c2 cells	nuclear swelling, DNA damage, mitochondrial dysfunction	Rharass et al., 2016
			C57BL/6J mice	ROS↑-autophagy damage	Li et al., 2014
			NRCMs, ARVMs	calpains↑-titin degradation	Lim et al., 2004
			Postnatal rat cardiac myocytes	BNIP3↑-COX1-UCP3 disruption-ROS↑- mitochondrial dysfunction	Dhingra et al., 2014
		Necroptosis	Mice	ROS↑-RIPK3 + phosphorylated-CaMKII-MTPT opening	Zhang T. et al., 2016
			Neonatal mouse ventricular myocytes; C57BL/6 mice	p38-MAPK↑-NF-κB↑-RIP1↑-RIP3↑-MLKL↑	Yu et al., 2020
		Pyroptosis	H9c2 cells; C57BL/6J mice	TLR4↑-NLRP3↑-caspase-1↑-IL1β↑, IL-18↑	Singla et al., 2019; Tavakoli et al., 2019
			HL-1 cardiomyocytes; C57BL/6J mice	BNIP3↑-caspase-3↑-GSDME↑	Zheng et al., 2020
			Neonatal rat ventricular cardiomyocytes (NRVCs); H9c2 cells; C57BL/6J mice	Drp1↑-NOX1↑, NOX4↑-NLRP3↑-caspase-1↑	Zeng et al., 2020
			Primary neonatal rat cardiomyocytes; H9c2 cells; Wistar rats	TINCR-lncRNA↑-IGF2BP1↑-NLRP3↑-caspase-3↑-GSDMD-IL1β↑, IL-18↑	Meng et al., 2019
			H9c2 cells; Rats	SIRT1↑-NLRP3↑-caspase-1↑-IL1β↑, IL-18↑	Sun et al., 2020; Zhai et al., 2020
		Ferroptosis	mice	Nrf2↑-Hmox1↑-Fe↑-mitochondrial damage-lipid peroxidation	Fang et al., 2019
			Male SD rats	TLR4↑, NOX4↑	Chen et al., 2019
	Cisplatin	Apoptosis	Cardiomyocytes; C57BL/6 mice	ROS↑-mitochondrial dysfunction, ER stress-caspase-3↑	Ma et al., 2010
			Male Albino rats	mitochondrial DNA injury, nuclear DNA infraction	El-Awady el et al., 2011
	Cyclophosphamide	Apoptosis	Male Wistar rats	sarcoplasmic reticulum dilatation, mitochondrial disruption, nuclear membrane invagination	Lushnikova et al., 2008
			H9c2; Wistar albino rats	acrolein-ROS↑, RNS↑	Nagi et al., 2011; Kurauchi et al., 2017
			Male Wistar albino rats	mitochondrial dysfunction-ATP↓-caspase-3↑	Refaie et al., 2020
			Male Wistar rats	ROS↑-TLR4↑-NF-κB↑	El-Agamy et al., 2017
			Female Wistar albino rats	DNA damage-Bcl2↑, caspase↑	Avci et al., 2017
	5 -Fluorouracil	Apoptosis	ARVMs	ROS↑, GSH↓-lipid peroxidation, MMP-caspase↑	Eskandari et al., 2015
		Autophagy	Human umbilical vein endothelial cells, Human colorectal cancer cells Human cardiac myocytes	autophagosome↑	Focaccetti et al., 2015
			ARVMs	lysosomal membrane leakiness- autophagy damage	Eskandari et al., 2015
	Arsenic trioxide	Apoptosis	Cardiac myocytes; Wistar rats	ROS↑, Ca^2+^↑	Raghu and Cherian, 2009
			H9c2 cells; Wistar rats	ROS↑-GSH↓, GPx↓, GST↓, SOD↓-lipid peroxidation, MMP↓	Varghese et al., 2017
			H9c2 cells	mitochondrial damage-ATP↓-caspase-3↑	Vineetha et al., 2015
			Male Hy-line chickens	trace elements disorder-mitochondrial damage- Bax caspase-3/8	Li et al., 2019
			ARVMs; Male SD rats	SERCA2a↓-ER stress-CHOP↑, caspase-12↑, GRP78↑	Zhang J.Y. et al., 2016
			Chickens	TNFα↑, NF-κB↑, COX-2↑, iNOS↑	Li et al., 2017
			NRVCs; Wistar albino rats	ROS↑, Ca^2+^↑-p38↑, JNK MAPK↑-NF-κB↑, IKK↑- PARP, caspase-3↑	Ghosh et al., 2009; Fan et al., 2013
			Human pluripotent stem cells induced cardiomyocytes	gammah2ax↑-DNA damage	Bao et al., 2019
		Autophagy	Culture HL-1 murine atrial cardiomyocytes	Parkin-mitophagy	Watanabe et al., 2014
			Hy-line chickens, carp	PI3K↑-Akt↑-mTORC1↓	Li S. et al., 2018; Zhao et al., 2019
	Trastuzumab	Apoptosis	NRVMs, ARVMs	Bax↑, Bcl-xS↑, Bcl-xL↓-MMP↓-ATP↓-caspase↑	Grazette et al., 2004
			Primary cardiomyocytes	ErbB2↓-DNA damage	Rohrbach et al., 2005
			ARVMs	neuregulin-1/ErbB2-phosphorylated Akt ↓	Sawyer et al., 2002
			C3H/HeJ mice	TLR4 -TNFα↑	Yousif and Al-Amran, 2011
			NRVCs	MDM2↓- p53↑	Singh et al., 2011
			Human primary cardiomyocytes	Beclin1↓, Atg 5-12/14↓-EebB1-Y845/ErbB2-Y1248 -ERK/mTOR/ULK-1-ROS↑, mitochondrial dysfunction	Mohan et al., 2016
	Sunitinib	Apoptosis	H9c2; Male C57BL/6NRj mice	ROS↑-mitochondrial damage	Bouitbir et al., 2019
			SD rats	miR-133A↑-phosphorylation of Ask1/MKK7/JNK↓	Cooper et al., 2018
		Autophagy	H9c2 cells	autophagy flux↑	Zhao et al., 2010
	Imatinib	Apoptosis	Primary cardiomyocytes; Mice	GATA4↓-Bcl-2↓, Bcl-xL↓	Maharsy et al., 2014
			H9c2 cells	Sab-JNK-ROS↑, mitochondrial damage-caspase- 3/7/9↑	Chambers et al., 2017
		Autophagy	Neonatal cardiomyocytes	autophagy block-lysosome↑, p62↑	Hu et al., 2012
	Nilotinib	Apoptosis	H9c2 cells	ROS↑, ATF4↑, CHOP↑-MMP↓, caspase-3↑	Lekes et al., 2016
			H9c2 cells	ER stress-JNK↑, phosphorylated Akt↓-phosphorylated GSK3β↓-Nox4/ROS↑	Yang et al., 2018
	Sorafenib	Apoptosis	Zebrafish	Raf-1/B-raf↓-MEK↓-ERK↓	Cheng et al., 2011
	Ponatinib	Apoptosis	NRCMs; Zebrafish	Phosphorylated Akt↓, ERK1/2↓-caspase-3↑	Singh et al., 2019
	Dasatinib	Necroptosis	CCC-HEH-2 human embryonic cardiac tissues	RIP1↑, RIP3↑-HMGB1↑	Xu et al., 2018
	Mitoxantrone	Apoptosis	H9c2 cells	ROS↑-Ca^2+^↑-MMP↓-ATP↓-caspase-3↑	Rossato et al., 2013
			Neonatal cardiomyocytes H9c2 cells	Top2β↓-DNA damage	Damiani et al., 2018
Antidiabetic drug	Rosiglitazone	Apoptosis	H9c2 cells	NAPHD↑, iNOS↑, SOD↓, GR↓	Mishra et al., 2014
			C57BL/6 mice	ROS↑-mitochondrial dysfunction	He et al., 2014
	Pioglitazone	Apoptosis	Wistar rats	Sphingomyelinase↑, ceramidase↑	Baranowski et al., 2007
			Primary cardiomyocytes	Bax↑, phosphorylated p53↑, phosphorylated vegfr-2↓, Akt↓, mTOR↓	Zhong et al., 2018
Antiviral drug	Zidovudine	Apoptosis	Rats	ROS↑, peroxynitrite↑-DNA breaks-NAD ^+^-ATP↓	Szabados et al., 1999
			Primary human cardiomyocytes	ROS↑-mitochondrial disruptions-caspase-3/7↑	Gao et al., 2011
			Mice	Fas/Fas L↑-caspase-3↑	Purevjav et al., 2007
		Autophagy	C2C12 myocyte cells	autophagy inhibition-MMP, ROS↑	Lin et al., 2019
		Necrosis	Primary human cardiomyocytes	Zidovudine-PARP↑	Gao et al., 2011
Teratogen	Cyclophosphamide	Apoptosis	Primigravida Swiss Webster mice	DNA fragmentation degradation-caspase-3↑	Mirkes and Little, 1998
			Swiss-Webster mice	p38-MAPK↑-caspases cascade reactions	Mirkes et al., 2000

### Anticancer Drugs

#### Doxorubicin (DOX)

##### Apoptosis

Enhanced production of ROS is recognized as the classic mechanism of DOX induced cardiomyocyte death. ROS consists of both free radicals and non-free radicals derived from oxygen, including superoxide anions (O_2_^–^), hydrogen peroxide (H_2_O_2_), hydroxyl radicals (OH^–^), ozone (O_3_) and singlet oxygen (^1^O_2_) (Zorov et al., 2014). DOX can be reduced to semiquinone by the endothelial nitric oxide synthase (eNOS) and triphosphopyridine nucleotide (NADPH) oxidase, which in turn leads to production of O_2_^–^, a major free radical that can produce other ROS, such as H_2_O_2_ and hydroxyl radicals (OH^⋅^) (Vasquez-Vivar et al., 1997). This is the main pathway by which DOX treatment generates ROS. Moreover, Deng et al. (2007) found that reactions between DOX and NADPH could produce superoxide in the absence of any enzyme activity, suggesting ROS production may be caused by the chemical interaction of DOX and NADPH. The generated ROS further induced DNA damage, especially DNA single-strand breaks. Previous studies showed that DOX treatment suppressed the DNA binding activity of GATA binding protein 4 (GATA4), an oxidant-sensitive transcription factor that plays an important role in transducing nuclear events (Kim et al., 2003). *In vitro*, DNA breaks activated nuclear poly ADP-ribose polymerase 1 (PARP-1) to induce the synthesis of poly-ADP-ribose and cause ATP consumption by subsequent glycohydrolase reactions and ATP conversion, leading to the collapse of heart energy metabolism (Mukhopadhyay et al., 2009). Scaffold protein Sirt6 was proved to be protective against DOX-induced DNA damage (Brito et al., 2016). In addition to DNA damage, DOX-induced intracellular ion disorder also contributes to ROS production.

A previous study demonstrated that under aerobic conditions, DOX could automatically combine with iron to form DOX-iron complex, which increased the contents of OH^–^ by self-reduction, contributing to subsequent lipid peroxidation through membrane interactions (Malisza and Hasinoff, 1995). Another study also discovered that the levels of iron and ROS were up-regulated in DOX-treated cardiomyocytes, which finally induced the mitochondrial apoptosis through caspase-3 activation and cytochrome C release (Childs et al., 2002). The participation of intracellular iron in the degradation of hypoxia-inducible factors (HIF) has been demonstrated (Peyssonnaux et al., 2008). Latter research indicated that iron/HIF signaling mediated the cardio-protective effect of dexrazoxane, the unique authorized protectant for DOX-induced cardiotoxicity (Spagnuolo et al., 2011). Paradoxically, DOX-induced ROS increased the synthesis of ferritin and mediated the protective effect of DOX against iron-induced cardiotoxicity (Corna et al., 2004).

In addition, topoisomerase 2 beta (Top2β) was found to involve in ROS formation during DOX treatment (Vejpongsa and Yeh, 2014). Topoisomerase 2 (Top2), consisting of Top2α and Top2β, played a crucial role in DNA replication, transcription, and repair (Wang, 2002). Top2 was considered a target for the anticancer effect of anthracyclines (Zhu et al., 2016). Top2β interaction with DOX caused DNA double-strand breaks and further triggered transcriptome changes in cardiomyocytes (Zhang et al., 2012). The DOX-Top2β combination may inhibit the transcription of peroxisome proliferator-activated receptor gamma coactivator-1 (PGC1) including PGC1α and PGC1β, which play critical roles in mitochondrial biogenesis as antioxidant (Finkel, 2006; Finck and Kelly, 2007). Moreover, mice with cardiomyocyte-specific Top2β conditional knockout (Top2β^–/–^) presented less DNA damage and mitochondrial dysfunction as well as oxidative phosphorylation after DOX exposure (Zhang et al., 2012).

Doxorubicin-induced oxidative stress can also stimulate death reporters to combine with corresponding cognate ligands, thereby inducing assembly of the DISC complex, which in turn caused caspase cascades activations and substrates cleavages. A recent study showed that the four death reporters (Fas, TNFR1, DR4, and DR5) were significant increased at the mRNA and protein levels after DOX treatment in induced pluripotent stem cells (iPS) -derived cardiomyocytes (Zhao and Zhang, 2017). One study suggested that ROS-induced up-regulation of cytosolic calcium concentration further elevated the expression of Fas Ligand (Fas L) by stimulating the nuclear factor of activated T-cells (NFAT) signaling (Kalivendi et al., 2005). Moreover, the calcium and calmodulin can conversely bind with eNOS electrons to increase superoxide formation, exacerbating cardiotoxicity (Vasquez-Vivar et al., 1998). Additionally, cardiac fibroblasts exacerbated cardiomyocyte apoptosis by releasing exosomes carrying Fas L in a paracrine manner during DOX treatment. Rosmarinic acid was shown to decrease Fas L secretion by suppressing the level of NFAT activation and metalloproteinase 7 (MMP7) expressions in cardiac fibroblasts, and served a protective role (Zhang et al., 2019). Preclinical experiments demonstrated a decrease in cardiomyocyte death of rats treated with anti-Fas L antibody (Nakamura et al., 2000). Moreover, caspase-8 inhibition blocked Fas L-induced apoptosis, indicating that downstream signaling of apoptosis was mediated by Fas L (Yamaoka et al., 2000). The matricellular protein CCN1 triggered by DOX reacted with integrin α_6_β_1_ to promote the activation of p38 mitogen-activated protein kinase (p38-MAPK), which stimulated the release of second mitochondrial activator of caspase (SMAC) and high-temperature requirement protein A2 (HtrA2), synergizing with Fas L to induce cardiomyocytes apoptosis (Hsu and Mo, 2016). As an important receptor in extrinsic apoptosis, TNFR1 was also involved in DOX-related cardiomyocyte death. DOX changed the level of TNFα in H9c2 cells, leading to an increase in TNFR1 expression and a decrease in TNFR2 expression, accompanied by the activation of caspase-8 and suppression of IκBα (Chiosi et al., 2007). It is noteworthy that vitamin C, a well-known reductant, can evidently decline the levels of TNFα, IL-1β and IL-6 in DOX-treated mice, indicating that oxidation/nitrosation stress may be one of the targets of cardiac protection during DOX treatment (Akolkar et al., 2017). In addition, the intracellular calcium homeostasis protective agent mangiferin was also verified to be able to relieve the up-regulation of TNFα and caspase-9 induced by DOX and stimulates the calcium regulatory gene, preventing myocarditis and apoptosis (Agustini et al., 2016).

Another plausible mechanism is that DOX-induced oxidative stress breaks the oxido-reduction balance of mitochondria in cardiomyocytes. DOX treatment promotes ROS overproduction, which remains inside the mitochondrial membrane and induces mitochondrial dysfunction (Giulivi et al., 1995). DOX caused a down-regulation of antioxidant enzymes such as copper, manganese and zinc superoxide dismutases (SODs), glutathione peroxidase (GSH-Px) and catalase (Costa et al., 2013). This imbalance between oxidation and antioxidation aggravates mitochondrial damage. Therefore, overexpression of antioxidant enzymes can reduce DOX-induced cardiotoxicity. *In vitro*, γ-ray pre-irradiation increased manganese superoxide dismutase (Mn-SOD) levels in neonatal rat ventricular myocytes (NRCMs), which up-regulated MMP, Bcl-2 expressions, and decreased the Bax expression and cytochrome C release (Childs et al., 2002; Chae et al., 2005). Nevertheless, polysaccharide elevated MMP and restrained mitochondrial permeability transition (MPT) by activating manganese superoxide dismutase (Mn-SOD) and suppressing the subsequent caspases cascade reactions (Li W. J. et al., 2018).

Recent studies demonstrated that DOX could indirectly target some receptors or anti-apoptosis factors by regulating miRNAs. DOX increased the level of miR-146a and induced the targeted inhibition of human epidermal growth factor receptor-4 (ErbB4), which caused cardiomyocyte apoptosis and acute cardiotoxicity (Horie et al., 2010). The miR-181a directly targeted the Bcl-2 transcript and negatively regulated Bcl-2 expression, which mediates the protective effect of propofol against DOX-induced cardiotoxicity *in vitro* and *in vivo* (Zhao et al., 2018). Moreover, miR-29b was found to target 3’ untranslated region of Bax and restrained Bax expression, hence alleviating DOX-induced cardiomyocyte apoptosis (Jing et al., 2018).

Several studies showed that varying DOX dosages caused apoptosis through different pathways. A study reported that treatment with a high concentration of DOX (2 μM) tended to promote ROS accumulation, while a lower concentration (0.25 M) was more likely to suppress the expression of haem oxygenase 1 (HO-1). HO-1 down-regulation induced cardiomyocyte apoptosis by activating caspase-3 and the release of mitochondrial cytochrome C (Bernuzzi et al., 2009). Another study found that a high concentration of DOX (1 μM) tended to cause DNA damage, PARP-1 dissociation and grievous apoptosis, and a low concentration of DOX (0.5 μM) could activate the p53-related mitochondrial apoptosis pathway (Cunha-Oliveira et al., 2018). Furthermore, DOX dose-dependently increased p53 expression in H9c2 cells, which inhibits type 1 insulin-like growth factor receptor (IGF-1R) transcription and induces IGF binding protein-3 (IGFBP-3) transcription, resulting in resistance to IGF-1 and contributing to apoptosis (Fabbi et al., 2015). More in-depth study indicated that the regulation of DOX on p53 may involve Sirtuin 1 (SIRT1) -mediated deacetylation of p53 (Zhang et al., 2011).

##### Autophagy

Autophagy is commonly considered as a conservative and beneficial regulatory process that maintains intracellular homeostasis, which is initially activated to resist DOX-induced cardiotoxicity. Oxidative stress is considered the main inducement for autophagy. As reported, during DOX treatment, ROS increased the ratio of LC3II/LC3I and the level of Beclin 1, both being the bio-markers of autophagy (Zhang et al., 2015). In addition, Dox up-regulated the levels of pro-autophagy factors (p53, p38-MAPK, and JNK-MAPK), and down-regulated the p85 expression, the catalytic subunit of phosphoinosmde-3-kinase (PI3K) as well as Akt phosphorylation (Ludke et al., 2017; Yu et al., 2017).

Even though the autophagy process is indeed initiated by DOX to serve a protective role, it somehow fails to finish the process since overwhelming oxidative stress blocks the degradation of lysosomes and even causes autophagic cell death, which in fact turns the original protective effect into damage. Under these circumstances, the normal protein degradation of cardiomyocytes was disrupted, and the subsequent increase in ubiquitinated proteins resulted in the accumulation of autophagy flux and autophagosomes (Dimitrakis et al., 2012). Meanwhile, DOX suppressed lysosome acidification and autolysosome degradation, which blocked the autophagic flux and augmented the damage (Li et al., 2016). Moreover, DOX-induced up-regulation of histone deacetylase 6 (HDAC6) decreased α-tubulin acetylation level, giving rise to mitochondrial dysfunction and autophagy flux damage (Song et al., 2018). Lysosome dysfunction was found to involve in the depletion of transcription factor EB (TFEB). DOX can suppress the expression of TFEB and induce the impairment of lysosomal cathepsin B, which subsequently inhibited lysosomal autophagy, increasing the levels of ROS and caspase-3 cleavage (Bartlett et al., 2016).

In addition to ROS-related autophagy, DOX also regulates autophagy-related factors and cause autophagic cell death. High mobility group box 1 (HMGB1) plays a vital role in the process of autophagy. DOX increased HMGB1expression, while silencing HMGB1 could reverse cardiomyocyte damage by attenuating autophagy (Luo et al., 2018). In addition, inhibition of the transcription factor GATA4 was observed in DOX-treated cardiomyocytes, and GATA4 induces the expression of Bcl2, which can interact with Beclin 1 to silence autophagy, decreases the cardiotoxicity (Kobayashi et al., 2010). Moreover, rats treated with 3-methyladenine, a specific inhibitor of autophagy, showed fewer autophagic vacuoles and mitochondrial MPT, but higher levels of Na^+^-K^+^ ATPase activity and MMP as compared with DOX treatment alone (Lu et al., 2009).

It has been reported that starvation or caloric restriction prior to DOX insult can suppress cardiotoxicity. Caloric restriction attenuated DOX-induced ATP exhaustion and enhances the activity of AMPK, which eventually corrected the harmful autophagy caused by DOX, demonstrating a protective role (Chen et al., 2011). On the contrary, prior starvation mitigated acute DOX-induced cardiotoxicity via further augmenting autophagy (Kawaguchi et al., 2012). In addition, Astragalus polysaccharide and resveratrol can restore autophagy in mice and H9c2 cells through the AMPK/mTOR signaling pathway, alleviating cardiotoxicity (Gu et al., 2016; Cao et al., 2017).

##### Necrosis

Unlike apoptosis and autophagy, emerging evidence has indicated that cardiomyocyte necrosis is triggered by a high dosage or prolonged exposure to DOX treatment. Dose-dependently elevated by DOX, the accumulation of ROS and peroxynitrite increase the rate of necrosis in cardiomyocyte death (Mukhopadhyay et al., 2009; Fulbright et al., 2015). The commonly used dosages of DOX are ≤20 mg/kg *in vivo* and 1 μM *in vitro*. A single intraperitoneal injection of DOX at 25 mg/kg in mice could immediately cause necrotic death and cardiac insufficiency (Li et al., 2014), and 2 μM DOX can directly induce cardiomyocyte necrosis *in vitro* (Bernuzzi et al., 2009). Moreover, when the cardiomyocytes are exposed to DOX for a long period, initial apoptosis develops into necrosis as the cells preferentially exhibits early DNA impairment and nuclear swelling (Rharass et al., 2016). As discussed above, DOX can destroy the function of lysosomes and disrupt normal autophagy as a result of oxidative stress. Consequently, the delayed autophagy in cardiomyocytes causes more severe apoptotic secondary necrosis (Dimitrakis et al., 2012; Li et al., 2014). These studies further confirm the notion that necrosis arose with extended exposure to DOX treatment.

Moreover, DOX initiates necrotic cell death by regulating necrosis-related intracellular factors. The degradation of titin, a myofilament protein associated with myocardial damage, was induced by DOX, and finally induced cardiomyocyte necrotic death (Lim et al., 2004). In addition, BH3-only protein BNIP3 was activated by DOX to destroy the combination of respiratory chain complex IV subunit 1 (COX1) and uncoupling protein 3 (UCP3), which disrupted respiratory efficiency, eventually leading to necrotic cell death (Dhingra et al., 2014).

##### Necroptosis

It has been demonstrated that RIPK3 is activated by DOX to bind with phosphorylated-CaMKII, causing the opening of mitochondrial permeability transition pore (MTPT), and resulting in necroptosis (Zhang T. et al., 2016). More importantly, dexrazoxane alleviated Dox-induced inflammation and cardiomyocyte necroptosis through inhibiting p38-MAPK/nuclear factor kappa-B (NF-κB) signal (Yu et al., 2020).

##### Pyroptosis

The increased secretion of IL-1β and IL-18, activation of TLR4, NLRP3 inflammasome and caspases were found in DOX-treated H9c2 cells, suggesting the occurrence of pyroptosis (Singla et al., 2019). BNIP3, the upstream regulator of cardiomyocyte pyroptosis, can activate caspase-3 and lead to subsequent GSDME cleavage (Zheng et al., 2020). DRP1/NOX signaling was activated to cause mitochondrial damage, which involved in DOX-induced pyrotosis (Zeng et al., 2020). Moreover, up-regulated lncRNA TINCR recruited IGF2BP1 to enhance the NLRP3 expression that mediated Dox-induced pyrotosis (Meng et al., 2019). However, embryonic stem cells-derived exosomes and Heat-shock Protein 22 can reverse the Dox-induced cardiomyocytes pyrotosis via inhibiting TLR4/NLRP3/caspase-1 signaling (Tavakoli et al., 2019; Lan et al., 2020). Moreover, suppression of ROS was also reported to be able to alleviate Dox-induced cardiomyocyte pyrotosis, whose mechanism involved the inhibition of sirtuin 1/NLRP3 signaling pathway (Sun et al., 2020; Zhai et al., 2020).

##### Ferroptosis

Doxorubicin-induced accumulation of ROS and lipid peroxidation can lead to cardiomyocyte ferroptosis (Koleini et al., 2019). Activation of TLR 4 and NOX 4 has also been proven to promote DOX-induced cardiomyocyte ferroptosis (Chen et al., 2019). Administering DOX to mice induced cardiomyopathy with a rapid, systemic accumulation of nonheme iron via heme degradation by NF-E2-related factor 2 (Nrf2)-mediated up-regulation of heme oxygenase-1 (HMOX1), indicating the cardio-protective role of targeting ferroptosis for cardiomyopathy prevention (Fang et al., 2019).

#### Cisplatin

Cisplatin is a chemotherapeutic agent for a vast spectrum of cancers. However, its acute and cumulative cardiotoxicity partially limits anti-tumor treatment and clinical applications (Ma et al., 2010). Cisplatin-treated cardiomyocytes showed mitochondrial abnormalities such as mitochondrial membrane depolarization, inflammatory responses and increased ER stress, which finally stimulated the activity of caspase-3 and induced apoptosis (Chowdhury et al., 2016). In addition, emerging evidences demonstrated a close connection between oxidative stress and cisplatin-induced cardiomyocyte apoptosis. El-Awady el et al. (2011) discovered that cisplatin improved lipid peroxidation, decreased GSH content and suppressed SOD activity, implying oxidative stress induced by cisplatin. Moreover, mitochondrial DNA injury and nuclear DNA damage were observed. Antioxidant natural products such as tutin (vitamin P1), zingerone and cyanidin can inhibit cisplatin-induced inflammatory infiltration, DNA damage, and mitochondrial dysfunction, indicating the key role of oxidative stress in cisplatin-induced cardiomyocyte apoptosis (Qian et al., 2018; Soliman et al., 2018; Topal et al., 2018).

#### Cyclophosphamide

Cyclophosphamide is commonly applied in the treatment of malignant tumors such as leukemia and lymphoma, and it is also adopted to treat systemic lupus erythaematosus and polymyositis as an immunosuppressor. Because of the dose-dependent manner, cyclophosphamide-induced cardiotoxicity basically coincides with high-dose treatment (Nishikawa et al., 2015; Wadia, 2015). Acrolein, the active metabolite of cyclophosphamide, was confirmed to be mainly responsible for cardiomyocyte death (Conklin et al., 2015; Nishikawa et al., 2015; Kurauchi et al., 2017). The cardiomyocyte injuries caused by cyclophosphamide treatment included sarcoplasmic reticulum dilatation, mitochondrial disruption and nuclear membrane invagination (Lushnikova et al., 2008). Further studies attributed these injuries to oxidative stress, clarifying that acrolein caused oxidative and nitrite stress through the suppression of intracellular GSH and SOD and increase of MDA (Nagi et al., 2011; Kurauchi et al., 2017; Omole et al., 2018). Corresponding lipid peroxidation initiated mitochondrial function damage, which further led to a collapse in APT production and the activation of caspase-3, resulting in apoptosis (Nagi et al., 2011; Refaie et al., 2020). In addition, cyclophosphamide was verified to stimulate TLR4, through which it initiated the TLR4/NF-κB signaling to trigger an inflammatory reaction, and eventually apoptosis (El-Agamy et al., 2017). Furthermore, DNA damage was observed in cyclophosphamide-treated rats, which were accompanied with activation of caspase-3 and inhibition of Bcl-2 expression (Avci et al., 2017). Antioxidant drugs such as silymarin and curcumin can inhibit cyclophosphamide-induced cardiotoxicity via decreasing the fragments of mitochondrial DNA and nuclear DNA, suggesting that excessive ROS might be responsible for cyclophosphamide-induced DNA injuries.

With the exception of the evidence discussed above, cyclophosphamide acted as a teratogen to injure cardiomyocytes. Cyclophosphamide affected cardiomyocytes developing via DNA fragmentation degradation, caspase-3 activation and PARP cleavage (Mirkes and Little, 1998). Moreover, cyclophosphamide was found to activate the apoptotic pathways, culminating in abnormality of the heart via the p38-MAPK signaling (Mirkes et al., 2000).

#### Fluorouracil and Capecitabin

5-Fluorouracil, a pyrimidine antimetabolite, is used widely in clinical practice as an anti-tumor treatment for cancers such as intestinal cancer and liver cancer. Capecitabin, a tumor-targeting drug that takes effect after the intracellular transformation into 5-fluorouracil, was regarded as having similar cardiotoxicity. Multiple studies confirmed the cardiotoxicity induced by 5-fluorouracil, which was attributed to the coronary arteries injury (Clavel et al., 1988; Herrmann et al., 2016). Further study suggested that the cardiomyocyte apoptosis might also play an important role in 5-fluorouracil-induced cardiotoxicity (Tsibiribi et al., 2006). 5-Fluorouracil stimulated intracellular oxidative stress by O_2_^–^ generation, which eventually activated the caspases cascade reactions, leading to apoptosis (Lamberti et al., 2012). To further determine the underlying mechanism, Eskandari et al. (2015) discovered that 5-fluorouracil-induced ROS increase was accompanied by the depletion of GSH, a ROS scavenger. Next, the generated ROS mediated lipid peroxidation on mitochondria to decrease MMP, causing mitochondrial dysfunction and caspase-3 activation. Fluoroacetate, the metabolite of 5-fluorouracil, can restrain aconitase to block the tricarboxylic acid cycle, resulting in a mitochondrial energy metabolism crisis (Lischke et al., 2015). Moreover, accumulation of autophagosomes and lysosomal membrane leakiness were observed in 5-fluorouracil-treated human cardiomyocytes, indicating the involvement of autophagic cell death in 5-fluorouracil-induced cardiotoxicity (Eskandari et al., 2015; Focaccetti et al., 2015).

#### Arsenic Trioxide

Arsenic trioxide represents a breakthrough in the field of acute promyelocytic leukemia therapeutic, but its cardiotoxicity remains an unresolved problem. In the past few decades, numerous studies have proposed that cardiomyocyte apoptosis is induced by ROS, and mitochondrial damage might be the main reason for arsenic trioxide-induced cardiotoxicity (Zhao et al., 2008; Raghu and Cherian, 2009; Vineetha et al., 2015). Arsenic trioxide can increase ROS level and calcium concentration, which was accompanied with cardiomyocyte apoptosis (Raghu and Cherian, 2009). Moreover, ROS generation depleted intracellular antioxidants such as GSH, GSH-Px, glutathione s-transferase (GST) and SOD, which caused lipid peroxidation and decreased MMP (Varghese et al., 2017). In addition, mitochondrial damage such as MTP and mitochondrial swelling was also observed under arsenic trioxide exposure, following by a decrease in oxygen consumption as well as ATP production, resulting in caspase-3 activation and apoptosis (Vineetha et al., 2015). Recently, Li et al. (2019) found that arsenic trioxide interfered with the dynamic balance of trace elements in chicken cardiomyocytes to break mitochondrial cristae and mitochondrial vacuoles, increasing the expressions of Bax and caspase-3/8. In addition to ROS and mitochondrial damage, calcium imbalance is also involved in arsenic trioxide-induced cardiotoxicity. Arsenic trioxide suppressed the activity of sarcoplasmic reticulum Ca^2+^-ATPase2a, by which cytoplasmic calcium was taken back to the sarcoplasmic reticulum (Zhang J.Y. et al., 2016). Consequently, the imbalance of calcium homeostasis and ER stress activated C/EBP-homologous protein (CHOP), caspase-12 and GRP78, leading to apoptosis.

By increasing the levels of the inflammatory cytokines TNFα, NF-κB, cyclooxygenase-2 (COX-2) and inducible nitric oxide synthase (iNOS), arsenic trioxides promoted the inflammatory reactions and brought ultrastructural damage to cardiomyocytes (Li et al., 2017). These inflammatory responses partly contributed to heavy metal-related cardiotoxicity (Lakkur et al., 2015). Additionally, it has been determined that arsenic trioxides up-regulates the content of phosphorylated p38 and JNK by oxidative stress stimulation and calcium overload, which further induces NF-κB phosphorylation, caspase-3 activation and PARP cleavage (Ghosh et al., 2009; Fan et al., 2013). Suppression of ROS apparently inhibited the activation of JNK, extracellular regulated protein kinases (ERK), and p38, which eventually reversed cardiomyocyte apoptosis (Miao et al., 2013; Zhang et al., 2017). One recent study reported that arsenic trioxides might induce cardiomyocyte apoptosis through DNA damage, since it dose-dependently increased the content of γH2AX, a sensitive biomarker for DNA breaks (Bao et al., 2019). Moreover, activation of Parkin, an E3 ubiquitin ligase, can inhibit arsenic trioxide-induced cardiotoxicity via the maintenance of mitochondrial as well as cellular homeostasis (Watanabe et al., 2014). It is well known that Parkin-induced ubiquitination of mitochondrial substrates finally leads to mitophagy. Therefore, mitophagy displays a positive role in maintaining cardiac homeostasis during arsenic trioxide exposure. However, a subsequent study demonstrated that arsenic trioxide-induced oxidative stress led to the formation of autophagosomes through PI3K/Akt/mTOR signaling, which resulted in myocardial damage (Li S. et al., 2018; Zhao et al., 2019). Moreover, cardiomyocyte necrosis was also observed under arsenic trioxide exposure (Raghu and Cherian, 2009; Vineetha et al., 2015), but the underlying mechanisms remained unclear.

#### Trastuzumab

A novel and widely used monoclonal antibody drug, Trastuzumab, is also reported to cause cardiotoxicity, such as cardiac insufficiency and heart failure. Under physiological condition, neuregulin-1 interacted with epidermal growth factor receptor-2 (ErbB2) to allow for the formation of ErbB4/ErbB2 heterodimer, which blocked cell death through an Akt-dependent signaling in cardiomyocytes (Sawyer et al., 2002; Lemmens et al., 2007). Functioning as an inhibitor of ErbB2 receptor, trastuzumab interrupted ErbB4/ErbB2 heterodimerization, thereby leading to apoptosis (Holbro and Hynes, 2004; Rohrbach et al., 2005). Interaction between Trastuzumab and ErbB2 triggered the downstream signal transduction pathways, such as increased levels of Bax and Bcl-xS, decreased Bcl-xL level and caspases cascade activations. Furthermore, the decreased MMP caused by Bcl-xL suppression and subsequent mitochondrial energy catastrophe also contributed to trastuzumab-induced apoptosis (Grazette et al., 2004). Singh et al. (2011) proposed that the cardiotoxicity induced by trastuzumab might stem from its negative regulation of murine double minute 2 (MDM2) and p53. In addition, trastuzumab-induced cardiomyocyte apoptosis was found to be related to inflammatory infiltration (Coppola et al., 2016), and the TLR4-mediated chemokine expressions of TNFα, MCP-1 and ICAM-1 contributed to inflammatory responses induced by trastuzumab (Yousif and Al-Amran, 2011).

A recent study reported that trastuzumab suppressed autophagy, resulting in mitochondrial dysfunction and ROS accumulation (Mohan et al., 2017). Trastuzumab insult inhibited the expressions of Beclin 1, autophagy related gene (Atg) 5-12 and Atg14. Moreover, Trastuzumab stimulated EebB1-Y845 and ErbB2-Y1248 and activated the ERK/mTOR/ULK-1 signaling to supress autophagy (Mohan et al., 2016).

Currently, drug combination therapies with trastuzumab are quite popular among cancer treatment plans. However, the combination therapies show more serious cardiotoxicity compared with single drug remedies. Trastuzumab combining with DOX exacerbated the exhaustion of antioxidant enzymes as well as damage to the mitochondrial structure (Xu et al., 2004). Trastuzumab-induced ErbB2 inhibition further suppressed c-Abl and Arg, which plays an antioxidant role by activating GSH-Px and catalase, thereby increasing DOX-induced cytotoxicity (Belmonte et al., 2015). Additionally, inhibition of the neuregulin-ErbB signaling by trastuzumab restrained the phosphorylation of ERK1/2 and Akt, and eventually exacerbated DOX-induced cardiomyocyte apoptosis and myocardial fiber injury (Sawyer et al., 2002). The underlying mechanism might also include the increased level of iNOS, which contributed to oxidative stress and inflammatory cell infiltration (Milano et al., 2020). Moreover, DOX-trastuzumab combination synergistically repressed Top2β, and eventually resulted in DNA double strand breaks and the ROS overproduction (Jiang et al., 2018). N-Acetyl Cysteine Amide, a ROS scavenger, can attenuate the DOX and trastuzumab-induced cardiac dysfunction (Goyal et al., 2016). Furthermore, paclitaxel-trastuzumab combination was reported to have a worsening effect on cardiomyocyte function, which might result from the inhibition of phosphorylated-ERK1/2. However, no cell death was observed during treatment in addition to myofibrillar structure changes (Pentassuglia et al., 2007).

#### Tyrosine-Kinase Inhibitors (Sunitinib, Imatinib, Nilotinib, Sorafenib, Ponatinib and Dasatinib)

Tyrosine-kinase inhibitors such as sunitinib, imatinib, nilotinib, sorafenib, ponatinib, and dasatinib are widely applied for chronic myelogenous leukemia and solid tumors, however, an increasing number of studies have reported cardiotoxicity associated with tyrosine-kinase inhibitors. Clinical research observed aberrantly shaped, swollen mitochondria in cardiomyocytes of patients who developed congestive heart after sunitinib treatment (Chu et al., 2007). Bouitbir et al. (2019) demonstrated that the oxidative stress caused by sunitinib was mainly responsible for the mitochondrial damage and final apoptosis of cardiomyocytes. By suppressing mitochondrial electron transport chain enzyme complexes, sunitinib induced ROS accumulation, which further decreased MMP and destroyed the mitochondrial structure to initiate caspases cascade reactions. In addition, sunitinib was confirmed to increase the expression of miR-133A, and suppress the apoptosis signal-regulating kinase 1 (ASK1)/mitogen activated kinase kinase 7 (MKK7)/JNK signaling to induce apoptosis and myocardial damage (Cooper et al., 2018). The elevation of autophagy flux was observed in sunitinib-treated H9c2 cells, (Zhao et al., 2010), and inhibition of autophagy was demonstrated to attenuate sunitinib-induced cardiotoxicity (Kimura et al., 2017), indicating involvement of autophagy in the cytotoxicity.

Mitochondrial damage was also observed in imatinib-treated cardiomyocytes. Imatinib down-regulates the expression of Bcl-2 and Bcl-xL by suppressing the content of the transcription factor GATA4, resulting in apoptosis. Moreover, aging was verified to be a risk factor during imatinib treatment because of its positive impact on the oxidative stress (Maharsy et al., 2014). In addition, Chambers et al. (2017) showed that imatinib induced oxidative stress and mitochondrial dysfunction via mediating JNK-related mitochondrial signaling and activating caspase-3/7/9. Autophagic death was also reported to be associated with imatinib-induced cardiotoxicity (Hu et al., 2012). Imatinib blocked the autophagic process as indicated by an increased level of lysosomes, which was consistent with the accumulation of p62, a protein degraded by autophagic clearance.

As a second-generation Bcr-Abl inhibitor, nilotinib mediated apoptosis mainly through the accumulation of ROS and ER stress (Doherty et al., 2013). The increase of ROS and ER stress biomarkers (ATF4 and CHOP), was observed in nilotinib-treated H9c2 cells, which subsequently decreased MMP and activated caspase-3, markers of apoptosis (Lekes et al., 2016). Recently, Yang et al. (2018) made further efforts to demonstrate that nilotinib induced ER stress to activate JNK and restrain Akt phosphorylation, which in turn suppressed glycogen synthase kinase-3 beta (GSK3β) phosphorylation and activated NOX4/ROS signaling, resulting in apoptosis.

Few studies reported potential apoptosis mechanisms associated with sorafenib, ponatinib and dasatinib. Cheng et al. (2011) demonstrated that sorafenib induced cardiomyocyte apoptosis and cardiotoxicity by inhibiting the Raf/MEK/ERK signaling. In addition, cardiomyocytes necrosis was observed during high-dose sorafenib treatment with unclear mechanism (Duran et al., 2014). The most recent study reported that ponatinib inhibited phosphorylation of Akt and ERK1/2, which contributed to the activation of pro-apoptotic caspase-3 (Singh et al., 2019). Xu et al. (2018) found that dasatinib dose-dependently up-regulated intracellular HMGB1 to induce cardiomyocyte necroptosis.

#### Mitoxantrone

With similar structure to DOX, mitoxantrone was thought to be an alternative to DOX with less cardiotoxicity (Herman et al., 1997). However, several studies have also reported mitoxantrone-induced cardiotoxicity. Mitoxantrone induced cardiomyocyte apoptosis associated with oxidative stress and mitochondrial dysfunction. With a time-dependent increase in the content of ROS, mitoxantrone promoted the accumulation of calcium and decreased the MMP, which further caused mitochondrial energy deficiency and activated caspase-3 (Rossato et al., 2013). In addition, DNA strand breaks were observed in apoptotic H9c2 cells treated with mitoxantrone, which was probably mediated by a Top2β-dependent signaling (Wu et al., 2013; Damiani et al., 2018).

#### Antidiabetic Drugs (Rosiglitazone and Pioglitazone)

Rosiglitazone and pioglitazone, as thiazolidinedione antidiabetic agents, were regarded as protective agents in diabetic cardiomyopathy and ischaemia-reperfusion injury (Cao et al., 2007; Baraka and Abdelgawad, 2010). However, with further expansion of their clinical applications, rosiglitazone and pioglitazone are also found to cause serious side effects such as myocardial hypertrophy and heart failure. It has been reported that rosiglitazone exerts both protective and detrimental effects in rats treated by ischaemia reperfusion (Palee et al., 2013). Although rosiglitazone was known as a PPARγ agonist, it caused cardiotoxicity via oxidative stress-induced mitochondrial dysfunction independent of PPARγ. By increasing the levels of NAPHD and iNOS, rosiglitazone induced oxidative stress accompanied by the exhaustion of antioxidant enzymes such as SOD and glutathione reductase, resulting in cardiotoxicity apoptosis (Mishra et al., 2014). In addition, the oxidative effect caused mitochondrial dysfunction, followed by cardiomyocyte energy deficiency (He et al., 2014). For pioglitazone, a study once reported that pioglitazone up-regulated the levels of sphingomyelinase and ceramidase, a mediator of cardiomyocyte apoptosis (Baranowski et al., 2007). Moreover, by activating Bax and phosphorylated p53 as well as suppressing phosphorylated Akt and mTOR, pioglitazone could induce apoptosis in a VEGFR-2 dependent manner (Zhong et al., 2018).

#### Antiviral Drug (Zidovudine)

Zidovudine, like other nucleoside reverse transcriptase inhibitors, is widely used for human immunodeficiency virus type 1 (HIV-1) infection. However, side effects such as hypertension and cardiomyopathy limit its long-term application. Zidovudine stimulated the accumulation of ROS and peroxynitrite, which in turn gives rise to single-strand DNA breaks, and eventually results in mitochondrial energy depletion in a NAD^+^-dependent manner (Szabados et al., 1999). Zidovudine induced the transport of protein kinase C δ (PKCδ) from the cytosol to the membrane, which promoted the activation of NADPH oxidases (Papparella et al., 2007). Meanwhile, mitochondrial damage is observed after zidovudine treatment (Fiala et al., 2004). Mitochondrial ROS caused by zidovudine played a significant role in mitochondrial disruption-induced apoptosis by activating caspase-3/7 (Ry et al., 2011). Fas/Fas L was also involved in zidovudine-induced cardiomyocytes apoptosis (Purevjav et al., 2007). Besides the above effects, zidovudine was shown to inhibit autophagosome maturation and decrease autophagic flux, leading to mitochondrial membrane polarization and ROS accumulation (Lin et al., 2019). Zidovudine-induced cardiomyocyte necrosis involved PARP activation (Gao et al., 2011).

## Perspectives and Conclusion

Cardiotoxicity is a major concern when evaluating whether drugs can be put on the market during preclinical research and is an important reason for post-approval drug withdrawal. Even for widely used drugs, cardiotoxicity limits their clinical applications. Fortunately, the mechanisms of cardiotoxicity have gradually come to light in recent years. ROS serves a main driver in drug-induced cardiotoxicity, and thereby many antioxidants have undergone preclinical development or clinically research for cardiotoxicity. For example, dexrazoxane, an iron chelating agent against iron-mediated oxidative stress, is the cardio-protective medicine approved by FDA in July 1995 for preventing anthracycline-induced cardiotoxicity, and now has been widely applied in the clinical practice (Padegimas et al., 2020). In addition, numerous natural antioxidants serve as adjuvant therapies to reduce drug-induced cardiotoxicity, such as berberine, epigallocatechin-3-gallate, and resveratrol (Coelho et al., 2017; Yu et al., 2018).

Recently, immune checkpoint inhibitors including anti-PD-1, anti-PD-L1 and CTLA-4 blockade have attracted a substantial amount of attention, which may revolutionize the treatment of cancer. Ever since the first case of cardiotoxicity induced by ipilimumab (CTLA-4 blockade) was reported in 2013 (Voskens et al., 2013), more and more case reports have indicated immune checkpoint inhibitor-induced cardiotoxicity. Worse still is the underlying mechanism remains unknown, possibly due to the lack of suitable animal models. Immune inflammation and ROS accumulation may play key roles in the immune checkpoint inhibitor-induced cardiotoxicity, which needs to be confirmed by future studies.

To achieve a better treatment effect, combinations of anticancer drugs have been widely applied in the clinical practice, but unfortunately lead to greater cardiotoxicity than with individual drug. Much attention has been paid to trastuzumab combined with DOX for treating women with ErbB2-positive breast cancer. Addition of trastuzumab to adjuvant DOX chemotherapy increases the incidence of cardiotoxicity, and few studies have been conducted to explore the underlying mechanisms, with iNOS or Top2β-mediated oxidative stress being the only acceptable mechanism (Jiang et al., 2018; Milano et al., 2020). To sum up, the mechanisms of drug-induced cardiomyocyte death are not absolutely independent, with the crosstalk and overlap of signaling pathways perplexing and complicating the cardiotoxicity. Therefore, further in-depth mechanisms deserve urgent investigation to avoid synergistic cardiotoxicity.

In this review, we summarized and discussed six cardiomyocyte death forms associated with drug-induced cardiotoxicity, including apoptosis, autophagy, necrosis, necroptosis, pyroptosis and ferroptosis. However, most of studies focused on the apoptosis, and whether the coexistence of multiple cardiomyocyte death forms was a common phenomenon of drug-induced cardiotoxicity remains to be explored. A recent study found that caspase-8 was the molecular switch that controls apoptosis, necroptosis and pyroptosis, and prevented tissue damage during embryonic development and adulthood (Fritsch et al., 2019). Therefore, it may be an interesting and important research topic to explore the contribution of each form and conversion of different forms in drug-induced cardiomyocyte death.

## Author Contributions

WM and SW wrote the manuscript. BZ and WL revised the manuscript. All authors read and approved the final version of the manuscript for publication.

## Conflict of Interest

The authors declare that the research was conducted in the absence of any commercial or financial relationships that could be construed as a potential conflict of interest.
